# ENHANCED COMMUNICATION BETWEEN A RURAL CRITICAL ACCESS HOSPITAL, A RESIDENTIAL CARE FACILITY, AND PRIMARY CARE

**DOI:** 10.1093/geroni/igac059.1338

**Published:** 2022-12-20

**Authors:** Lee Morissette

**Affiliations:** Mount Ascutney Hospital, Windsor, Vermont, United States

## Abstract

A need to improve the transitions of care between a small rural emergency department, a residential care facility and a primary care practice emerged early on in our work to become a level 2 accredited emergency department. One challenge is bringing together 3 partners all with distinct types of health records, unique foci of care and a variety of disciplines and workforce. This paper will focus on our journey of creating an individualized plan of care to help mitigate problems of poor communication with a focus on the 4Ms framework including: What Matters Most, Mobility, Medication and Mentation.

